# Event-Related Potential Responses to Task Switching Are Sensitive to Choice of Spatial Filter

**DOI:** 10.3389/fnins.2018.00143

**Published:** 2018-03-08

**Authors:** Aaron S. W. Wong, Patrick S. Cooper, Alexander C. Conley, Montana McKewen, W. Ross Fulham, Patricia T. Michie, Frini Karayanidis

**Affiliations:** ^1^Functional Neuroimaging Laboratory, School of Psychology, University of Newcastle, Callaghan, NSW, Australia; ^2^Priority Research Centre for Stroke and Brain Injury, University of Newcastle, Callaghan, NSW, Australia; ^3^Priority Research Centre for Brain and Mental Health, University of Newcastle, Callaghan, NSW, Australia; ^4^Department of Psychiatry, Center for Cognitive Medicine, Vanderbilt University Medical Center, Nashville, TN, United States

**Keywords:** Laplacian, REST, common average, average mastoids, cognitive control, ERP, switch cost, mixing cost

## Abstract

Event-related potential (ERP) studies using the task-switching paradigm show that multiple ERP components are modulated by activation of proactive control processes involved in preparing to repeat or switch task and reactive control processes involved in implementation of the current or new task. Our understanding of the functional significance of these ERP components has been hampered by variability in their robustness, as well as their temporal and scalp distribution across studies. The aim of this study is to examine the effect of choice of reference electrode or spatial filter on the number, timing and scalp distribution of ERP elicited during task-switching. We compared four configurations, including the two most common (i.e., average mastoid reference and common average reference) and two novel ones that aim to reduce volume conduction (i.e., reference electrode standardization technique (REST) and surface Laplacian) on mixing cost and switch cost effects in cue-locked and target-locked ERP waveforms in 201 healthy participants. All four spatial filters showed the same well-characterized ERP components that are typically seen in task-switching paradigms: the cue-locked switch positivity and target-locked N2/P3 effect. However, both the number of ERP effects associated with mixing and switch cost, and their temporal and spatial resolution were greater with the surface Laplacian transformation which revealed rapid temporal adjustments that were not identifiable with other spatial filters. We conclude that the surface Laplacian transformation may be more suited to characterize EEG signatures of complex spatiotemporal networks involved in cognitive control.

## Introduction

Cognitive control processes support the ability to flexibly adapt to changing contextual demands by coordinating the integration of goal-appropriate neural and cognitive resources (Diamond, 2013). The task-switching paradigm is used to experimentally manipulate proactive and reactive cognitive control processes (Monsell, 2003; for a review see, Jamadar et al., 2015). In cued-trials task-switching paradigms, participants alternate between two or more 2-choice decision tasks using cues that on each trial reliably signal the need to change or repeat task. Performance is characterized by a switch cost, i.e., poorer performance on trials where the task changes as compared to trials where the task repeats and a mixing cost, i.e., poorer performance on repeat trials that are interspersed with switch trials (mixed-task block) compared to a block of repeat trials alone (single-task block). These performance costs are believed to arise from different contextual demands on the cognitive control system. For instance, switch cost is often attributed to the need to proactively or reactively adjust to changing task demands by resetting, whereas mixing cost to the need to maintain the relevant task goals in working memory. When the cue-target interval (CTI) is sufficiently long, performance costs reduce, indicating the engagement of proactive cognitive control processes (Braver, 2012) that prepare the system to update the new task-set or maintain the same task-set. However, a substantial residual switch cost remains even with long CTIs, indicating that reactive cognitive control processes are required for interference control even under highly prepared conditions. The excellent time resolution of event-related potentials (ERP) makes them a highly sensitive tool in characterizing proactive and reactive control processes involved in switching between tasks. Long CTI conditions can temporally dissociate cue-locked ERPs associated with preparation to switch or repeat from target-locked ERPs associated with implementation of the relevant task set.

When examining switching costs, cue-locked ERP waveforms show a robust differential *switch positivity* that manifests as a larger positivity for switch than repeat cues in mixed-task blocks extending around 200–600 ms after cue onset (e.g., Nicholson et al., 2006a; Lavric et al., 2008; Karayanidis et al., 2009; Barceló and Cooper, 2018). This switch positivity is most often maximal over parietal electrodes, although it typically spreads across the scalp (for review see Karayanidis et al., 2010) and has occasionally also been reported at frontal sites (Rushworth et al., 2002; Barceló et al., 2006; Astle et al., 2008; Barceló and Cooper, 2018). A number of other ERP differences between switch and repeat trials have also been reported during the CTI, including early and late frontal negativities and late centrally maximal negative shifts (see Karayanidis et al., 2010). Target-locked ERPs are also modulated by task-switching, with the most robust effect being a larger posterior P3b for repeat compared to switch trials (e.g., Astle et al., 2006, 2008; Nicholson et al., 2006b; Jamadar et al., 2010b).

Electrophysiological indices of the mixing cost have also been reported. In cue-locked ERPs, repeat trials in mixed-task blocks show larger frontal negativity (Goffaux et al., 2006; Manzi et al., 2011; Czernochowski, 2015) and centroparietal positivity (Jost et al., 2008; Manzi et al., 2011; Whitson et al., 2014) compared to repeat trials in single-task blocks. Following target onset, ERPs show smaller parietal P3b amplitude for repeat trials in mixed-task blocks than in single-task blocks (Goffaux et al., 2006; Whitson et al., 2014). Taken together, ERPs in task-switching paradigms have shown modulation of a number of anterior and posterior positivities as well as frontal negativities associated with task-switching costs.

These electrophysiological indices of task-switching costs are consistent with evidence from functional magnetic resonance imaging (fMRI) studies that frontoparietal brain networks are involved in implementation of cognitive control (Ruge et al., 2013). However, the lack of consistency in the number and spatial distribution of both cue-locked and target-locked ERP components across different studies and paradigms, makes it difficult to map the process-specificity of these ERP effects.

### The influence of spatial transformations

Even with modern EEG systems that record from 64 to 256 electrode sites, the spatial resolution of the EEG signal is inherently low. Moreover, the distribution of the EEG signal recorded at the scalp is highly sensitive to the type of spatial transformations that is applied to the EEG data, including the choice of reference configuration. Most commonly, ERP studies use a reference derived from the average of the signal recorded at the left and right mastoid processes or earlobes, a nasal electrode or a common average reference (e.g., the average signal across all electrode sites). Kayser and Tenke (2015) demonstrated that common average, average mastoids and nasal reference schemes produced subtle but complex differences in the timing, amplitude, polarity and distribution of ERP components.

The majority of ERP studies on task-switching use either common average or average mastoid reference while linked-ear (Goffaux et al., 2006) and nasal-weighted reference montages are less frequently employed (e.g., Jost et al., 2008; Kieffaber et al., 2013). In an early review of the ERP task-switching literature, Karayanidis et al. (2010) noted that the choice of a common average or an average mastoid reference configuration may contribute to discrepancies in the occurrence of early and late frontal switch effects. Despite the dramatic impact that the choice of reference configuration may have on the morphology of the ERP waveform, the effect of reference montage on ERPs elicited in task-switching paradigms has not been systematically investigated.

The cue-locked centroparietal switch positivity has been reported with both common average reference (Astle et al., 2006, 2008; Swainson et al., 2006; Lavric et al., 2008; Travers and West, 2008; Li et al., 2012; Capizzi et al., 2015; Lange et al., 2015) and average mastoids reference (Rushworth et al., 2002, 2005; Barceló, 2003; Miniussi et al., 2005; Barceló et al., 2006, 2008; Nicholson et al., 2006a,b; Karayanidis et al., 2009, 2011; Periáñez and Barceló, 2009; Jamadar et al., 2010a; Gajewski and Falkenstein, 2011; Hsieh and Wu, 2011; Manzi et al., 2011; Cunillera et al., 2012; Nessler et al., 2012; Tarantino et al., 2016; Barceló and Cooper, 2018). However, in cue-locked ERPs, a frontal switch positivity (Astle et al., 2008) and a frontal switch negativity (Poulsen et al., 2005; Astle et al., 2006; Lavric et al., 2008; Travers and West, 2008; Li et al., 2012; Capizzi et al., 2015) have been reported most consistently when using a common average reference (but see Rushworth et al., 2002; Barceló et al., 2006; Barceló and Cooper, 2018; for average mastoid effects using the intermittent instructions cued-trials design).

Discrepancies in the pattern of ERP findings as a function of reference montage have also been reported in mixing costs. For example, mixing cost effects are found at frontal, frontocentral and centroparietal electrodes when using average mastoids (Manzi et al., 2011; Czernochowski, 2015; Tarantino et al., 2016) and linked-ear references (Goffaux et al., 2006), but only centroparietally with a nasal reference (Jost et al., 2008). As cognitive control processes involved in task switching are thought to rely on extensive frontoparietal networks (e.g., Sauseng et al., 2006; Cooper et al., 2015; see also Kim et al., 2012), the choice of reference montage may impact on whether ERP waveforms capture the network dynamics underpinning cognitive control.

### Use of “reference free” spatial transformations

While a reference electrode is *sine qua non* for *recording* voltage potentials with EEG, when analyzing ERPs this reference can be changed arbitrarily. Ideally, entirely removing the confounding influence of a reference choice should improve the specificity (spatial, temporal or otherwise) of scalp-recorded neural activity. In recent years, two such *reference-free* transformation schemes have gained popularity: The reference electrode standardization technique (REST) or infinity reference scheme (Yao, 2001) and the surface Laplacian transformation (or current source density, Kayser and Tenke, 2006, 2015; Nunez and Srinivasan, 2006).

REST approximates a reference-free transformation for scalp-recorded electrical activity by computing a “reference at infinity.” This is derived using a distributed source model of cortical activity within a head model. In effect, the source model is fit to the observed data and then the model is used to compute the scalp-recorded EEG referenced to a neutral point at infinity. The rationale behind this approach is that the source-level EEG activity is independent of the choice of reference at the scalp: i.e., the generators of EEG signals within cerebral tissue are not dependent on the arrangement of recording electrodes placed on the head. Only the recorded signal depends on the reference choice and the REST procedure is intended to correct for this (for a technical review, see Yao, 2001).

Scalp-recorded EEG potentials are assumed to be associated with cortically generated electric current that flows radially outwards through the cranium and dissipates across the scalp. The radial outflow of cortical current is referred to as current source density, CSD (or alternatively, scalp current density; see Yao, 2002 for further explanation) and can be estimated using a surface Laplacian transformation (Hjorth, 1975). Most current implementations of this approach begin by interpolating the EEG scalp topography across electrodes using a surface spline. The Laplacian of a surface spline can be computed efficiently. Importantly, CSD is independent of the original choice of reference electrode. The surface Laplacian also acts as a high pass spatial filter, attenuating the effects of volume conduction such that it enhances sensitivity to focal activity in the cortical mantel, while suppressing widespread EEG signals originating from deeper layers (Kayser and Tenke, 2006). As such, the surface Laplacian is insensitive to broad changes in signal, resulting from volume conduction and reference choice, and more sensitive to activity from cortical generators. This results in improved spatial and temporal information (for a technical review, see Hjorth, 1975; Yao, 2002; Kayser and Tenke, 2015).

### Present study

Some task-switching ERP components show a similar spatial distribution across different reference schemes (e.g., cue-locked centroparietal switch positivity) whereas others vary as a function of reference choice (e.g., cue-locked frontocentral switch negativities), an observation previously made by Karayanidis et al. (2010). Despite concerns that the ERP components elicited during task-switching may vary with reference configuration, the effect of different references on ERPs related to task-switching has not been systematically examined. Moreover, *reference-free* spatial transformations that can improve the spatial localization of neural activity recorded at the scalp have not been widely used. No task-switching ERP study has used the REST transformation. A few studies have applied the surface Laplacian transformation only to ascertain that the switch effects found in average mastoid referenced data were not the result of volume conduction (Gajewski et al., 2010; Gajewski and Falkenstein, 2011; Barceló and Cooper, 2018). In this study, we directly compare the effect of four reference transformations on cue-locked and target-locked ERP data recorded using a cued-trials task-switching paradigm. We use two conventional references commonly used in task-switching studies that have produced some discrepant results: average mastoids and common average reference. We compare these to the two more recent approaches that are designed to reduce the impact of choice of reference electrode and volume conduction: REST and surface Laplacian. We aim to demonstrate that reference-free spatial transformations may help identify and differentiate between distinct ERP components associated with task switching.

Given that the posterior cue-locked switch positivity is robust across different standard referencing schemes, we expect that a large switch positivity will be evident in all four spatial transformations, and will show a strong midline parietal-occipital focus with surface Laplacian. Cue-locked frontal switch positivity and negativity effects have typically been reported only when using a common average referencing scheme, suggesting that this frontal component is sensitive to spatial transformation. If these frontal switch effects are an artifact of volume conduction in the common average transformation, they will be absent in the surface Laplacian transformation. Alternatively, if these switch effects represent distinct cognitive processes generated in frontal sources, they will be evident in both REST and surface Laplacian transformations.

Cue-locked mixing cost effects are also strongly linked to the choice of spatial transformation. Thus, this centroparietal component is unlikely to be due to volume conduction and should therefore be evident in all spatial transformations, including REST and surface Laplacian. In contrast, frontal and frontocentral effects are only seen for mastoid (or linked-ear) transformations. As before, if these frontal/frontocentral effects are the result of volume conduction, we expect to see no frontal mixing costs in the surface Laplacian transformation. If they represent activity originating from the frontal lobe, they will be present for both REST and surface Laplacian transformations.

With averaged mastoids, target-locked ERPs typically produce a sustained parietal negative shift in the ERP waveform for switch compared to repeat trials overlapping the N2 and P3b period. Reduced volume conduction with surface Laplacian may indicate whether this represents a broadly distributed component that overlaps the target-locked ERP or differential modulation of N2 and P3b components.

## Methodology

### Participants

Two hundred and thirty eight community participants took part in the current study as part of the larger Age-ility project (Karayanidis et al., 2016; http://www.age-ility.org.au/). Written, informed consent was obtained from all participants prior to testing, with parental consent also given for persons under 18 years of age. All participants were reimbursed $20 per hour for their time and were directed to abstain from caffeine and alcohol for at least 2 h prior to the experimental session. The protocol was approved by the University of Newcastle Human Research Ethics Committee (HREC: H-2012-0157).

In order to ensure strong signal-to-noise for ERP analyses, participants who had fewer than 50 trials on any trial type after EEG preprocessing were removed (*n* = 37). This resulted in a final sample of 201 participants (mean age 21.32 ± 4.91 years, 91 male, 91.5% right handed).

### Task and stimuli

**Figure 1:**
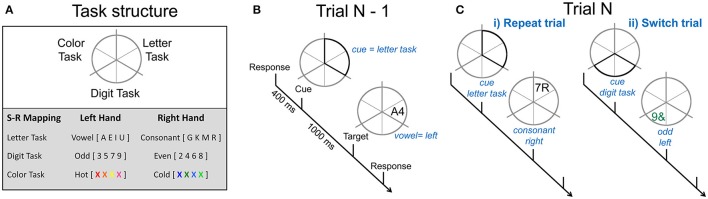
Cued-trials task-switching paradigm. **(A)** Adjoining segments were associated with different 2-choice classification tasks. An example of stimulus-response mappings is shown. **(B)** Example of one trial. A cue occurs 400 ms after the response to the preceding trial and highlights two segments, in this instance, related to the letter task, indicating that the next target will occur in one of these segments and will require a vowel vs. consonant decision. A target replaced the cue after 1,000 ms. Participants respond to the task-appropriate feature of this target. **(C)** The sequence of N-1 to N trials determines whether the current trial is a (i) repeat trial. i.e., the same two segments are highlighted and the same task is performed, or (ii) switch trial, i.e., the cue highlights segments associated with one of the other two tasks and validly indicates which task to perform on the target.

On each trial, participants performed a cued two-choice response task involving either the letter, digit or color features of the stimuli. Throughout each block of trials, a gray circle (5° of visual angle) was continuously displayed. The circle was divided into six segments with pairs of adjacent segments allocated to one of the three tasks: letter, digit, and color (see Figure 1A). The target was a pair of characters consisting of combinations of a letter, a digit, or a non-alphanumeric symbol and was presented either in gray or in color. Each target (e.g., gray A4) consisted of three dimensions: one relevant to the currently cued task (e.g., the letter A mapped to a left-hand response), one selected randomly from the two alternative tasks and incongruently mapped with the relevant task (e.g., the digit 4 mapped to a right-hand response) and one that was neutral, i.e., not mapped to any response (e.g., letter and digit presented in gray). The same target could not appear on consecutive trials and targets remained onscreen until a response was emitted or for 5,000 ms.

Each trial consisted of a cue-target sequence (see Figure 1B). Different cue locations resulted in four cue types that varied in information regarding the task to be performed on the target. In this paper, we restricted analyses to the fully informative cue types in order to focus on switch cost and mixing cost effects. All-repeat trials refer to trials derived from single-task blocks (i.e., where the same task was repeated on all trials in a block). Switch and repeat trials were derived from mixed-task blocks (i.e., trials on which the location of the cue fully identified the task to be performed on the next target). Thus, in both single-task and mixed-task blocks, repeat cues highlighted the same two segments as on the preceding trial (see Figure 1C), thereby indicating the task would be repeated. Switch cues highlighted two segments associated with one of the two tasks not completed on the previous trial. Contrasts were derived for switch cost (switch—repeat trials) and mixing cost (repeat—all-repeat trials).

Participants responded using their left and right index fingers. The hand assigned to each response was counterbalanced across participants. Participants were instructed to respond as quickly and as accurately as possible. A feedback tone was presented only when responses were incorrect. At the end of each block, average accuracy and RT were displayed and a short, humorous video (5–10 s) was presented to encourage inter-block rests. Participants were offered a longer rest halfway through the experiment to minimize fatigue.

### Procedure

Participants completed two training sessions (circa 14 days apart) to establish strong cue-stimulus-response links. Within each training session, participants first learned each task alone (single-task blocks) and then practiced switching between these tasks (mixed-task blocks, total of 1,320 practice trials). Following the second training session, the EEG was recording while participants performed 10 mixed-task blocks (72 trials/block) and three single-task blocks (48 trials/block, one block per task). Each block included an additional five warm-up trials.

EEG was recorded continuously using an ActiveTwo Biosemi EEG system (2,048 Hz, bandpass filter of DC-400Hz) from 64 scalp electrodes plus external electrodes at left and right mastoids and outer canthi, as well as supraorbital, and infraorbital ocular sites. Common mode sense (CMS) and driven right leg (DRL) electrodes were positioned inferior to P1 and P2, respectively. EEG data were recorded relative to an amplifier reference voltage, and then re-referenced offline to Cz in order to remove common-mode signals.

### Data analysis

Trials were removed from behavioral and EEG analyses if the trial (i) had an RT faster than 200 ms or slower than three SD above the individual's mean RT, (ii) was associated with an incorrect response or followed an incorrect response, or iii) was one of the warm-up trials at the beginning of each block. On average, 18% ± 7.6% of trials were removed. Trials with high EEG noise levels (see below, EEG Analysis, for more details) were also excluded from behavioral and EEG analyses.

#### Behavioral analysis

Mean RT and accuracy were assessed using separate one-way ANOVAs on the *trial type* factor (i.e., *all-repeat, repeat* and *switch*). Planned comparisons were performed to assess *switch cost*, (*switch*—*repeat*) and *mix cost* (*repeat*—*all-repeat*). Family-wise error Bonferroni correction was applied to RT and accuracy comparisons separately (i.e., α < 0.05/2) with Greenhouse-Geisser corrections for the assumption of sphericity.

#### EEG analysis

EEG data were processed using MATLAB (MATLAB, 2015) through a pipeline utilizing Fieldtrip (Oostenveld et al., 2011), REST software (from http://www.neuro.uestc.edu.cn/rest/; Yao, 2001) and CSD Toolbox (Kayser and Tenke, 2006) and in-house functions (A. Wong and P. Cooper). Preprocessing was performed using Fieldtrip as follows. EEG data were re-referenced off-line to electrode Cz, down-sampled from 2,048 to 512 Hz using a zero-phase anti-aliasing filter with a low-pass cut off frequency of 245 Hz and then had high pass and notch filtering applied to remove line noise and low-frequency drift (high pass: 0.1 Hz, forward phase; 50 Hz notch: zero phase). Excessively noisy channels were identified with visual inspection and excluded (on average 0.84 ± 1.59 electrode channels were removed per participant). For each cue type (*switch, repeat, all-repeat*) epochs were extracted from −1,000 to 3,500 ms with respect to cue onset. To remove blink and vertical eye-movement artifact, independent components analysis (ICA) was performed using the fastICA algorithm, (Hyvärinen and Oja, 2000). This produces a set of components, one less than the amount of available electrodes. Based on visual inspection by a trained observer, 1.40 ± 0.80 components were removed because they corresponded to ocular artifact (i.e., a deflection consistent with the time course of an eyeblink coupled with a frontal topographical distribution) The remaining components were projected back into sensor (electrode) space. The data were low pass filtered (30 Hz, zero-phase) to remove high frequency noise including muscular artifacts. Trials that contained residual artifact larger than ±120 μV were deleted, resulting in an average of 111.62 (±22.32 SD) *all-repeat*, 132.65 (±26.75 SD) *repeat*, and 127.29 (±28.66 SD) *switch* trials per participant for further analysis.

After preprocessing, EEG data were re-referenced using two commonly employed referencing schemes: *average mastoids* (i.e., the algebraic average of the mastoids) and *common average* (i.e., the average of all scalp electrodes). In addition, the *infinity referencing* transformation was applied using the REST software to obtain a reference-free EEG dataset. Finally, the *surface Laplacian* transformation was computed. For the *surface Laplacian*, a spherical spline function was applied across all scalp electrode locations, with the spline flexibility parameter, *m* = 4, for increased rigidness (Kayser and Tenke, 2015). An iterative process was used to solve a Legendre differential equation to obtain the surface Laplacian and surface potential matrices (Kayser and Tenke, 2006). As the EEG signal is transformed based on the second partial derivate of the signal (μV) over a spatial area (cm^2^–i.e., the scalp), the measurement scale is μV/cm2 (Kayser and Tenke, 2006, 2015).

#### ERP analysis

For each participant, epochs spanning from 200 ms before to 1,200 ms after event onset were extracted around the cue and the target, using a ±50 ms peri-event baseline. ERP waveforms were extracted by averaging across all trials for each cue type, and grand average ERPs were obtained by averaging across all participants for each cue type, separately for cue-locked and target-locked epochs.

As ERP activity related to task-switching is commonly larger at midline electrodes, we analyzed switch cost and mixing cost contrasts at frontal (Fz), central (Cz), and parietal (Pz) electrodes using paired sample *t*-tests using false discovery rate for type 1 errors (FDR, Benjamini and Yekutieli, 2001; α < 0.001). Intervals of significant activity were defined, if this level of significance was held for 25 ms or 13 consecutive time points, sampled at 512 Hz. These analyses were conducted on waveforms derived from each spatial transformation separately.

The intersection of these significant time points across all spatial transformations was derived to identify effects that were common across all transformations. For each common interval, the effect size, Hedges' g, of the cost at each spatial filter were also obtained and reported. These common intervals were used to examine the scalp distribution map of switch cost and mixing cost effects across different spatial transformations. For each scalp distribution map, we examined the spatial distribution of significant switch and mixing effects using a paired sample *t*-test (FDR, α < 0.001) at each electrode site.

## Results

### Behavioral results

**Figure 2:**
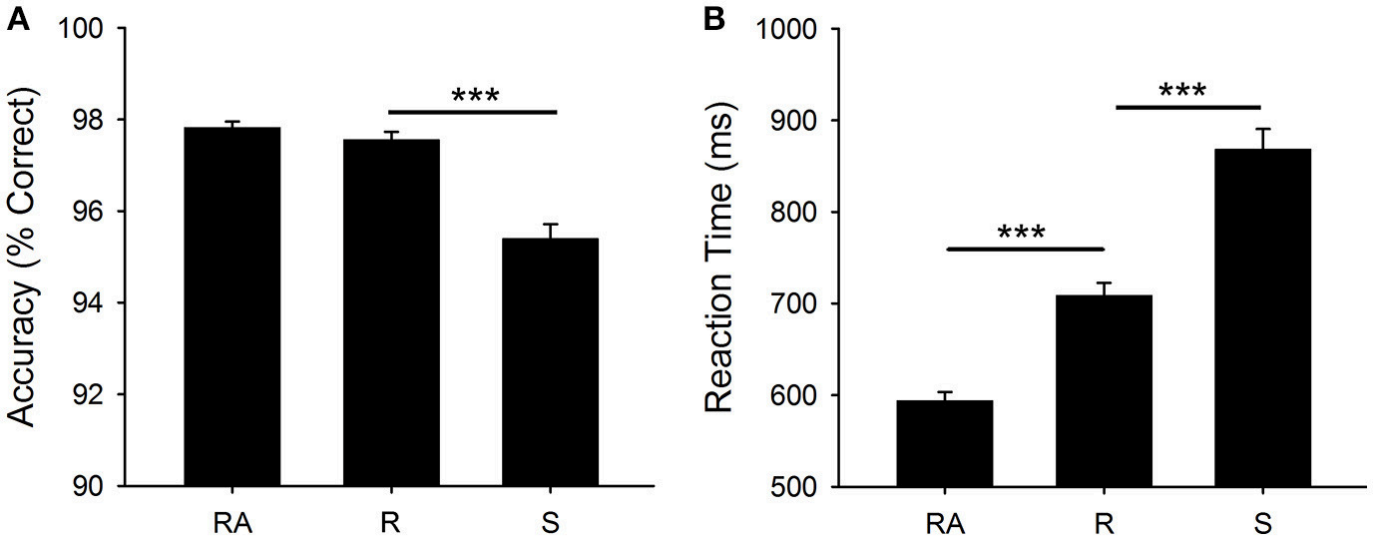
Behavioral Results: **(A)** Mean accuracy (percentage correct) and **(B)** Mean RT (ms). Significant differences are shown at ^***^p < 0.001. RA, *all-repeat*; R, *repeat*; and S, *switch*.

Figure 2 shows mean RT and accuracy for each cue type [*F*_(2, 400)_ = 263.9, *p* < 0.001, η^2^ = 0.569, *F*_(2, 400)_ = 75.0, *p* < 0.001, η^2^ = 0.273, respectively]. There was a significant mixing cost effect for RT [*t*_(200)_ = 14.9, *p* < 0.001] and a significant switch cost for both RT [*t*_(200)_ = 14.0, *p* < 0.001] and accuracy [*t*_(200)_ = 10.0, *p* < 0.001].

### Cue-locked ERP analyses

**Figure 3:**
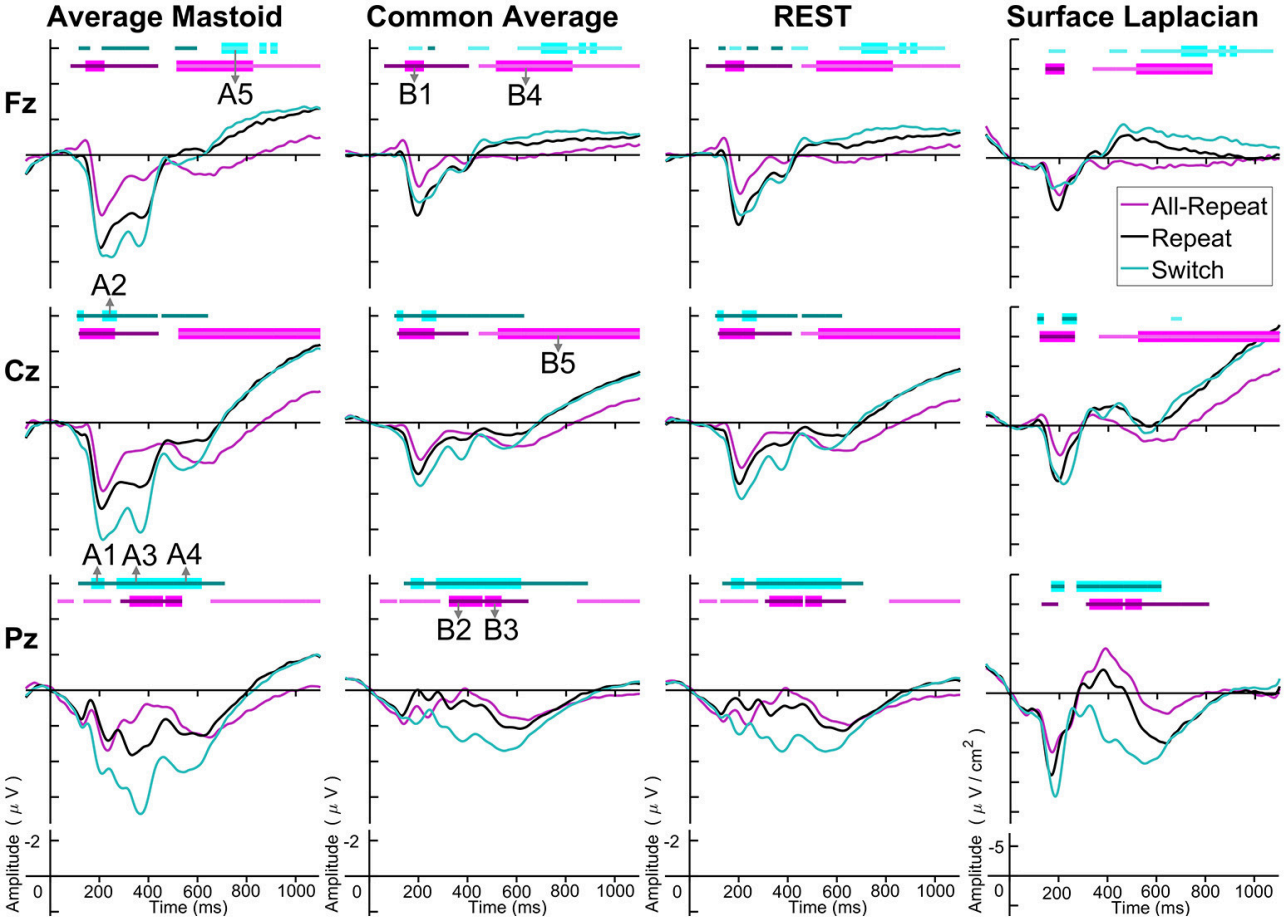
Cue-locked grand average ERPs for each spatial transformation at the three midline electrodes. All-repeat = magenta; repeat = black; switch = cyan. Significant intervals of switch or mix cost (FDR α < 0.001) are shown as thin cyan and magenta bars, respectively, with darker bars indicating positive costs (i.e., switch > repeat, repeat > all-repeat) and lighter bars indicating negative costs (i.e., reverse contrasts). Thick bars represent intervals over which the effect was significant for all spatial transformations. The scalp distribution of common effects for switch cost (A1-A5) and mixing cost (B1-B5) are shown in Figure 4. Note that average mastoid, common average and REST use the same amplitude scale in microvolts, whereas surface Laplacian uses microvolts/cm^2^. Gray line at 1,000 ms denotes target stimulus onset.

Figure 3 depicts cue-locked ERP waveforms for *all-repeat, repeat* and *switch* at three midline electrodes for each reference type. At first glance, there are substantial differences in global morphology across different transformations. However, as discussed below, there are many commonalities in both switch cost and mixing cost effects.

#### Switch costs

Both average mastoid and common average references produced a pattern of result very consistent with prior studies using these reference configurations. For average mastoids, the most prominent effect was a large parietally maximal switch positivity over roughly 120–600 ms that extended across all midline sites. This switch positivity was also evident with the common average reference, but was more restricted parietally, evident in Pz but not Cz nor Fz, whereas frontally there emerged a small sustained late switch negativity over 600–1,000 ms. This switch negativity was smaller and more brief with the average mastoid reference, however, it still reached statistical significance in this very large sample. REST showed a pattern remarkably similar to common average reference. The surface Laplacian showed the same effects but much more temporally and spatially defined. Specifically, the switch positivity was differentiated into an early brief centroparietal effect superimposed on the P2 and later parietally-restricted effect over 250–600 ms. The frontal switch negativity first emerged around 400 ms and then extended to the end of the epoch.

We identified intervals that produced significant switch effects across all transformations (see thick significance intervals in Figure 3), in order to compare their scalp distribution. This resulted in five intervals: a very early switch positivity over the parietal P2 (A1: 165–215 ms), a second effect most clearly evident at tail end of P2 centroparietally (A2: 215–275 ms), early and late windows to define the parietal switch positivity “proper” (A3: 270–350 ms, A4: 350–550 ms), and the frontal switch negativity (A5: 700–800 ms). Table 1 shows effect sizes for these of common cue-locked switch intervals for each transformation. With the exception of the frontal switch negativity, most intervals showed large effect sizes.

**Table 1:** Hedge's g effect size for common intervals of significant switch cost and mixing cost effects in cue-locked ERP waveforms shown in Figure 3.

**Cue-locked ERPs**					
**Figure 3 label (electrode)**	**A1 (Pz)**	**A2 (Cz)**	**A3 (Pz)**	**A4 (Pz)**	**A5 (Fz)**
**SWITCH COST**					
Interval (ms)	165–215	215–270	270–350	350–550	700–800
Average mastoids	0.69675	**0.61711**	**0.78378**	0.7158	−0.14975
Common average	**0.71151**	0.54026	0.68418	**0.79799**	−0.25344
REST	0.68465	0.566	0.72001	0.7826	–**0.29526**
Surface laplacian	0.43806	0.40959	0.40991	0.78949	−0.21915
**Figure 3 label (electrode)**	**B1 (Fz)**	**B2 (Pz)**	**B3 (Pz)**	**B4 (Fz)**	**B5 (Cz)**
**MIXING COST**					
Interval (ms)	150–220	330–450	470–530	540–800	800–1000
Average mastoids	0.59228	**0.65469**	0.22783	–**0.40918**	−0.58697
Common average	**0.84222**	0.28965	0.26836	−0.39057	−0.62036
REST	0.80191	0.38942	0.23567	−0.40876	–**0.63154**
Surface laplacian	0.32606	0.34368	**0.38611**	−0.34382	−0.4957

*Bold effect sizes denotes the largest of the four reference transformations*.

**Figure 4:**
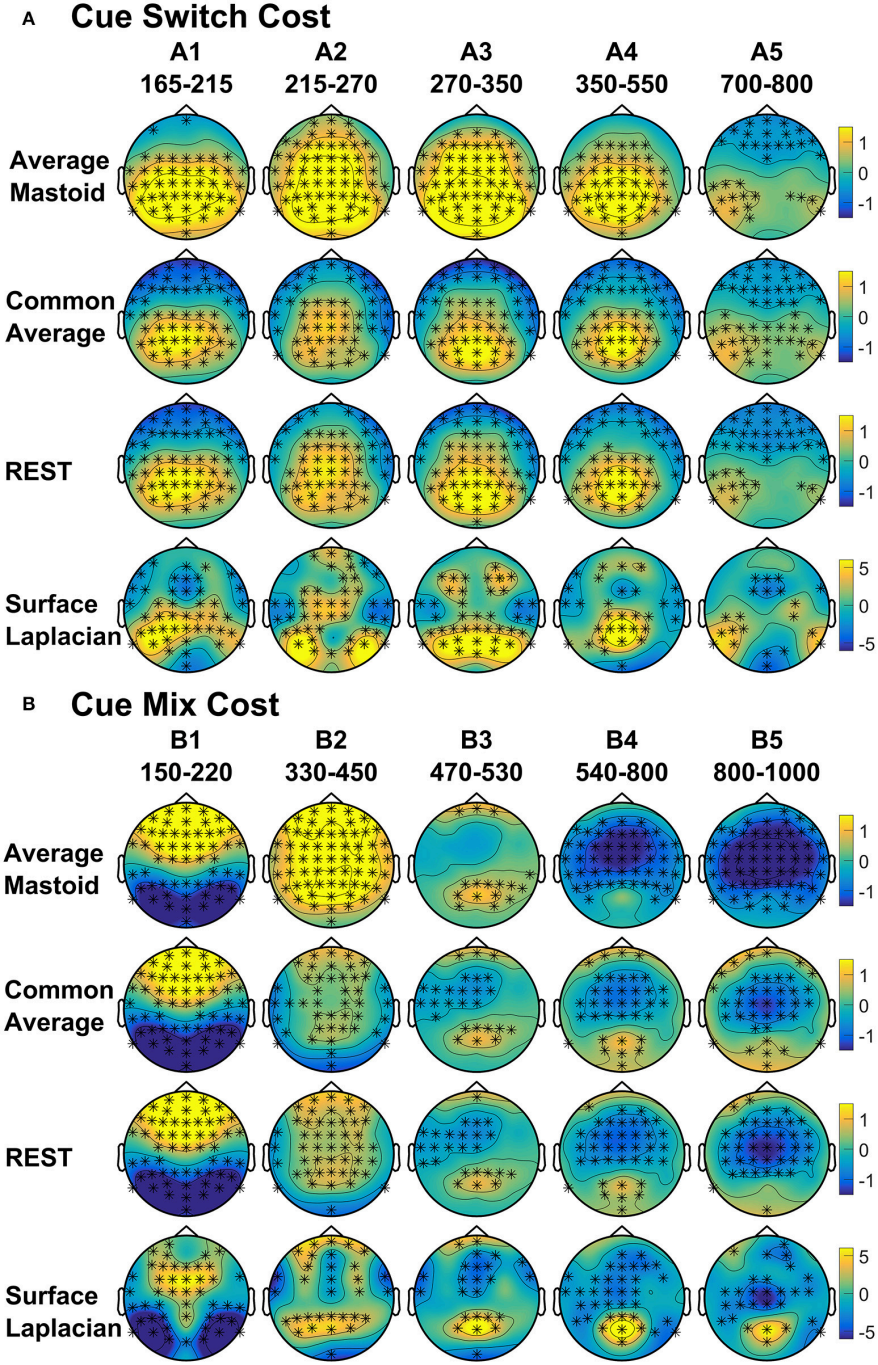
Scalp topography of cue-locked **(A)** Switch costs and **(B)** Mix costs for each spatial transformation depicted for the intersection of significant time intervals across all spatial transformations. Electrodes with significant differences (FDR α < 0.001) are marked with an asterisk. Note that average mastoid, common average and REST use the same amplitude scale in microvolts, whereas surface Laplacian uses microvolts/cm^2^.

As shown in Figure 4A, the average mastoids reference shows a switch positivity that spread across the first four intervals A1-A4 (i.e., 165–550 ms) and had a very broad scalp distribution with a centroparietal focus. Common average and REST references produced a similar centroparietally maximal switch positivity, however REST reference was less spatially distributed when compared to average mastoids and was accompanied by a concurrent frontal switch negativity. In contrast, the scalp distribution of the switch positivity varied over A1 to A4 in the surface Laplacian reference. There was an early bilateral parietal effect (A1) that shifted posteriorly and anteriorly (A2, A3) before developing a tight parietoccipital focus (A4). The late frontal switch negativity was present across all referencing schemes (A5), but was much more localized over the frontal midline for surface Laplacian. Thus, average mastoids, common average and REST indicate the presence of two temporally widespread components: a broadly distributed switch positivity and a later frontal switch negativity. However, the surface Laplacian is suggestive of a much more complex pattern of temporal and spatial dynamics associated with preparation to switch vs. preparation to repeat a task-set.

#### Mixing costs

The averaged mastoids reference showed the typical pattern of mixing effects consisting of an early mixing positivity followed by a late pretarget mixing negativity spread across midline electrodes (Figure 3). These effects were also significant with common average and REST references, albeit smaller and more variable (e.g., note the early mixing negativity at Pz). Once again, while the same pattern of effects was evident with surface Laplacian, the mixing effects were better defined both temporally and spatially.

The intersection of significant effects in Figure 3 across different spatial configurations was used to define five intervals: an early frontocentral mixing positivity (B1: 150–220 ms), a later parietal mixing positivity that was split into two windows (B2: 330–450 ms, B3: 470–530 ms), a slow frontal mixing negativity (B4: 540–800 ms) and a central ramp-like mixing negativity (B5: 800–1,000 ms). Table 1, show mostly medium size effects, with no clear indication of a superior reference choice across all time intervals.

Figure 4B shows that, as with switch cost effects, the average mastoids reference produce broadly distributed effects that were more spatially confined with common average and REST references and even further with surface Laplacian. Specifically, average mastoids suggest two temporally separable components: a frontocentral mixing positivity (B1) that spread across the scalp (B2) and dissipated mostly by 450 ms, and a centrally maximal mixing negativity that emerged around 500 ms and extended to the end of epoch. The surface Laplacian suggests a somewhat more complex pattern, differentiating more clearly between the early mixing effect that was characterized by a midline frontal positivity and bilateral posterior negativities (B1), a sustained parietoccipital mixing positivity spreading to the end of the epoch (B2–B5), and a frontocentral midline mixing negativity that emerged as early as 330 ms and increased in strength across the remainder of the epoch, as it overlapped the pretarget negativity (B2–B5). Scalp maps showed a frontal to central negative focus, accompanied by an occipital midline positivity, possibly suggesting of a parietal dipole.

### Target-locked ERP analyses

**Figure 5:**
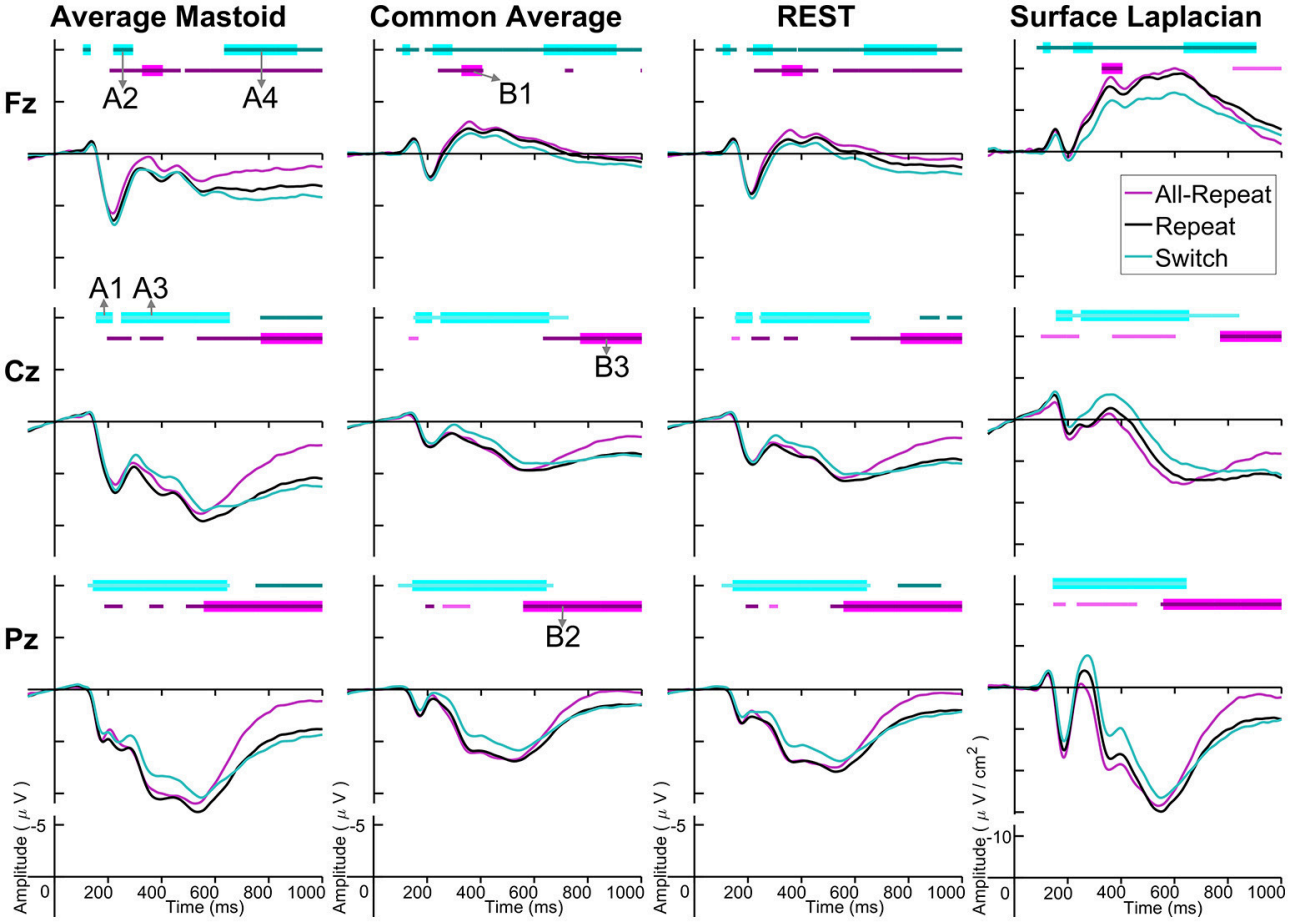
Target-locked grand average ERPs for each spatial transformation at three electrodes. All-repeat = magenta; repeat = black; switch = cyan. Significant intervals of switch or mix cost (FDR α < 0.001) are shown as thin cyan and magenta bars, respectively, with darker bars indicating positive costs (i.e., switch > repeat, repeat > all-repeat) and lighter bars indicating negative costs (i.e., reverse contrasts). Thick bars represent intervals over which the effect was significant for all spatial transformations. The scalp distribution of common effects for switch cost (A1-A4) and mixing cost (B1-B3) are shown in Figure 6. Note that average mastoid, common average and REST use the same amplitude scale in microvolts, whereas surface Laplacian uses microvolts/cm^2^.

Target-locked ERP waveforms depicted in Figure 5 are characterized by a large parietally maximal P3b that partially overlapped a centrally maximal N2 for all spatial transformations.

#### Switch costs

Averaged mastoid and common average references show a pattern consistent with previous work, including a broad parietal negative shift for switch as compared to repeat cues extending over 200–600 ms. This is typically interpreted as either a broad negativity superimposed on the N2-P3b complex or a reduced P3b. REST showed a very similar pattern to the common average reference. However, the morphology of the ERP waveform was very different for surface Laplacian. Parietally, there was a very well defined N1, P2, N2, P3 sequence and a switch negativity that clearly spread from early P2 to late P3b. This switch negativity was also evident at Cz where it overlapped a broad N2-P3b complex. However, both the waveform morphology and the switch effect were very different at Fz. Here, the waveform shows a large sustained negative wave from 200 ms and that is smaller for switch trials. Careful inspection of Fz shows that the reversed switch effect was evident for all other references (though less temporally widespread with average mastoids), but the effect was much smaller.

**Figure 6:**
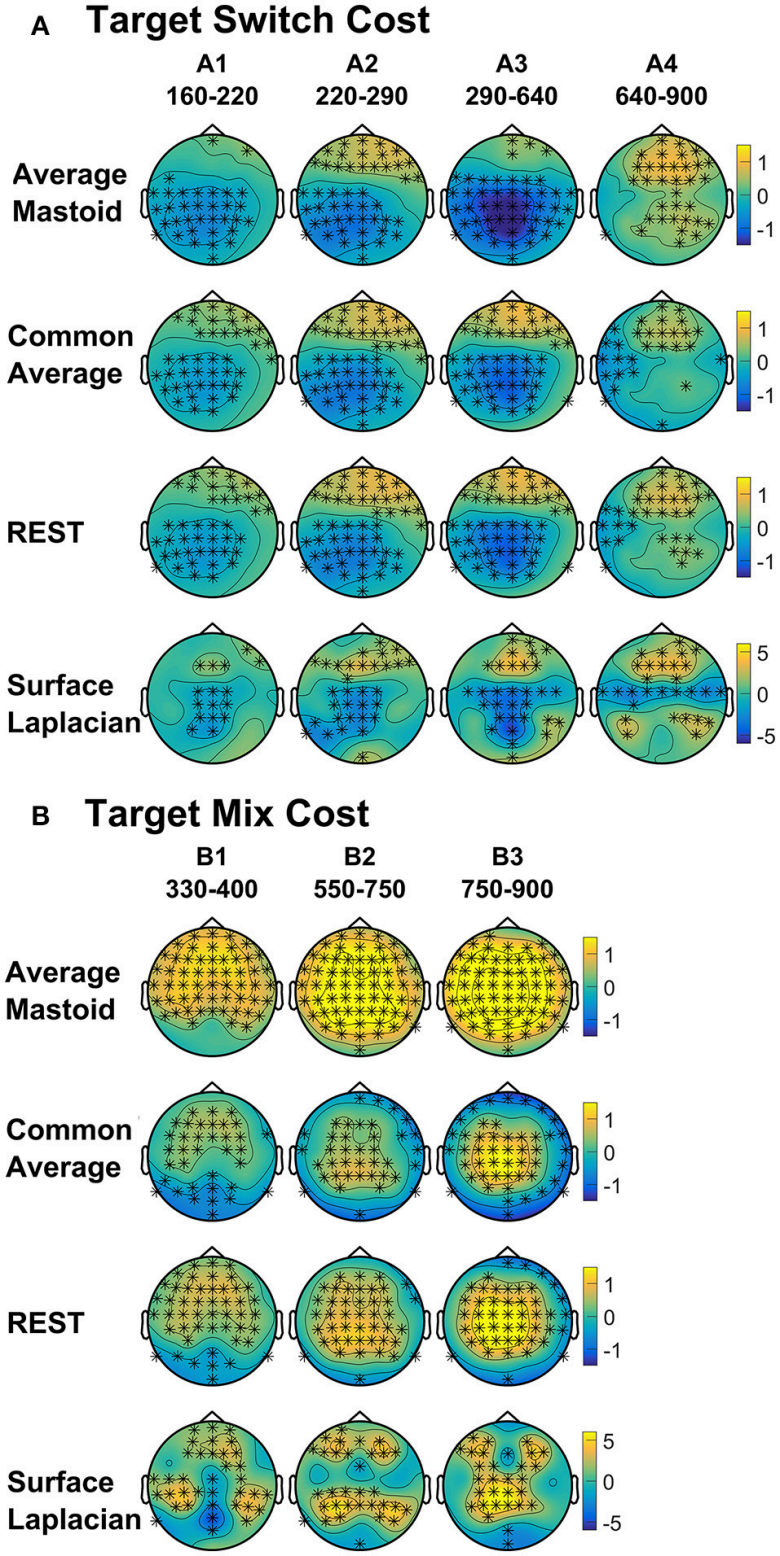
Scalp topography of target-locked **(A)** Switch costs and **(B)** Mix costs for each spatial transformation depicted for the intersection of significant time intervals across all spatial transformations. Electrodes with significant differences (FDR α < 0.001) are marked with an asterisk. Note that average mastoids, common average and REST use the same amplitude scale in microvolts, whereas surface Laplacian uses microvolts/cm^2^.

Four intervals were defined for topographical analyses: An early centrally maximal switch negativity (A1: 160–220 ms), a frontally maximal switch positivity (A2: 220–290 ms), a long centroparietal switch negativity (A3: 290–640 ms) and a late slow frontal switch negativity (A4: 640–900 ms). Table 2 shows small to medium effect sizes that were largest for common average and surface Laplacian transformations at different time intervals. In Figure 6, all four reference schemes show a very consistent centroparietal switch negativity spanning across A1-A3 (160–640 ms), but becoming increasingly spatially localized in common average, REST and surface Laplacian, compared to averaged mastoids. All four reference schemes also show a frontal switch positivity spreading across A2–A4, with a midline focus in surface Laplacian.

**Table 2:** Hedge's g effect size for common intervals of significant switch cost and mixing cost effects in target-locked ERP waveforms shown in Figure 5.

**Target-locked ERPs**				
**Figure 5 label (electrode)**	**A1 (Cz)**	**A2 (Fz)**	**A3 (Cz)**	**A4 (Fz)**
**SWITCH COST**				
Interval (ms)	160–220	220–290	290–640	640–900
Average mastoids	−0.17474	0.17268	−0.28326	0.24711
Common average	−**0.23927**	0.34019	–**0.32397**	0.27201
REST	−0.18199	0.29828	−0.27831	0.31543
Surface laplacian	−0.22777	**0.45722**	−0.30336	**0.32762**
**Figure 5 label (electrode)**	**B1 (Fz)**	**B2 (Pz)**	**B3 (Cz)**	
**MIXING COST**				
Interval (ms)	330–400	550–750	750–900	
Average mastoids	0.59228	**0.65469**	0.22783	
Common average	**0.84222**	0.28965	0.26836	
REST	0.80191	0.38942	0.23567	
Surface laplacian	0.32606	0.34368	**0.38611**	

*Bold effect sizes denotes the largest of the four reference transformations*.

#### Mixing costs

Mixing cost effects were also widespread for the averaged mastoids (Figure 5). A mixing positivity first emerged frontally around 200 ms, extending across to the end of the epoch. The effect emerged later centroparietally (^~^600 ms). The common average reference emphasized the late centroparietal mixing positivity, whereas the REST more closely resembled average mastoids. Surface Laplacian also showed the late centroparietal mixing positivity, but this was preceded by a centroparietal mixing negativity with little frontal differential activity. Intersection analyses defined three mixing positivities for topographical analyses: an early frontal (B1: 330–400 ms), a parietal (B2: 550–750 ms) and a late centroparietal (B3: 750–900 ms). The effect size of these effects were largest with average mastoids reference for three intervals (Table 2). Scalp maps for average mastoids show a temporally and spatially extended mixing positivity across all windows (Figure 6B). The effect was similar for common average and REST, but more centrally localized. However, the surface Laplacian showed a pattern of effects that varied with time and spatial location. The early frontal midline mixing positivity was accompanied centroparietally by bilateral positive and midline negative mixing effects (B1) that morphed into a late centroparietal mixing positivity, spreading bilaterally more frontally.

### Surface laplacian

**Figure 7:**
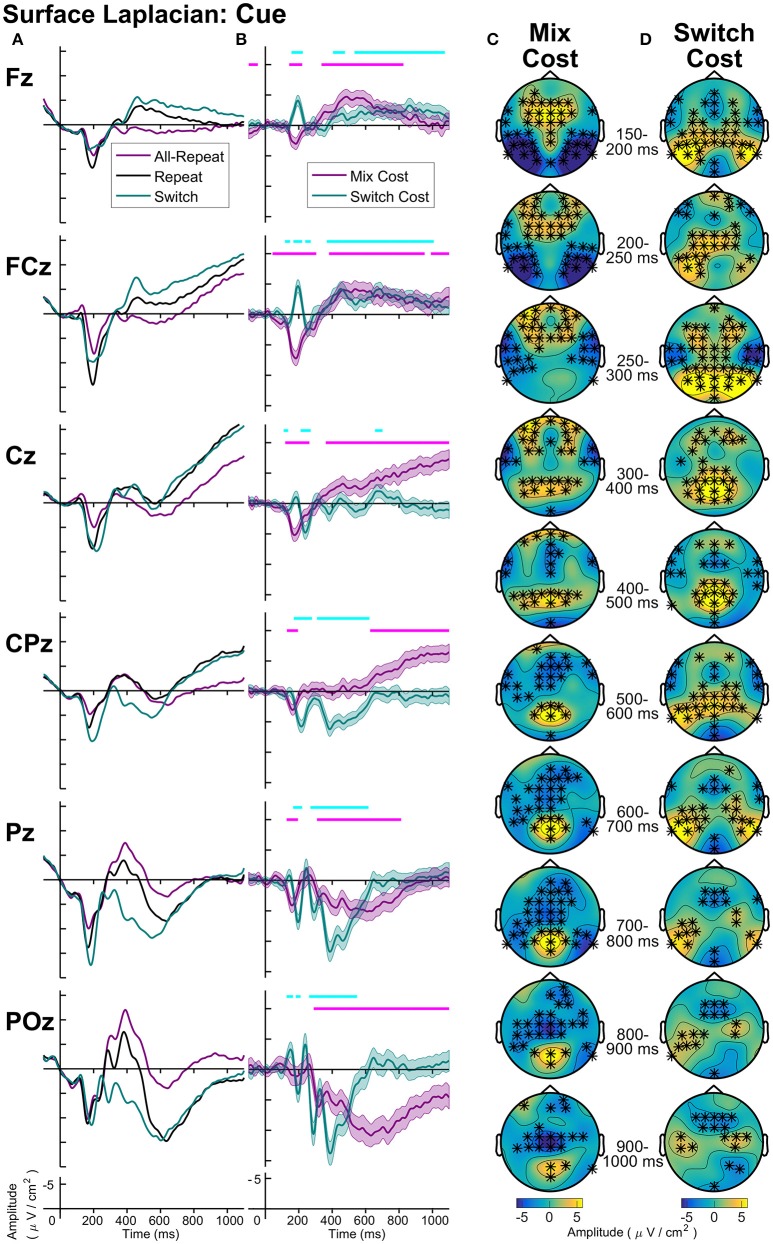
Surface laplacian cue-locked: **(A)** Grand average ERPs, **(B)** Difference waveforms (Costs), Shaded 95% confidence intervals calculated for a within-subjects design (c.f., Loftus and Masson, 1994), Significant intervals of switch or mix cost (FDR α < 0.001) are shown as thin cyan and magenta bars, respectively, **(C)** Mix Cost Topographies and **(D)** Switch Cost Topographies.

**Figure 8:**
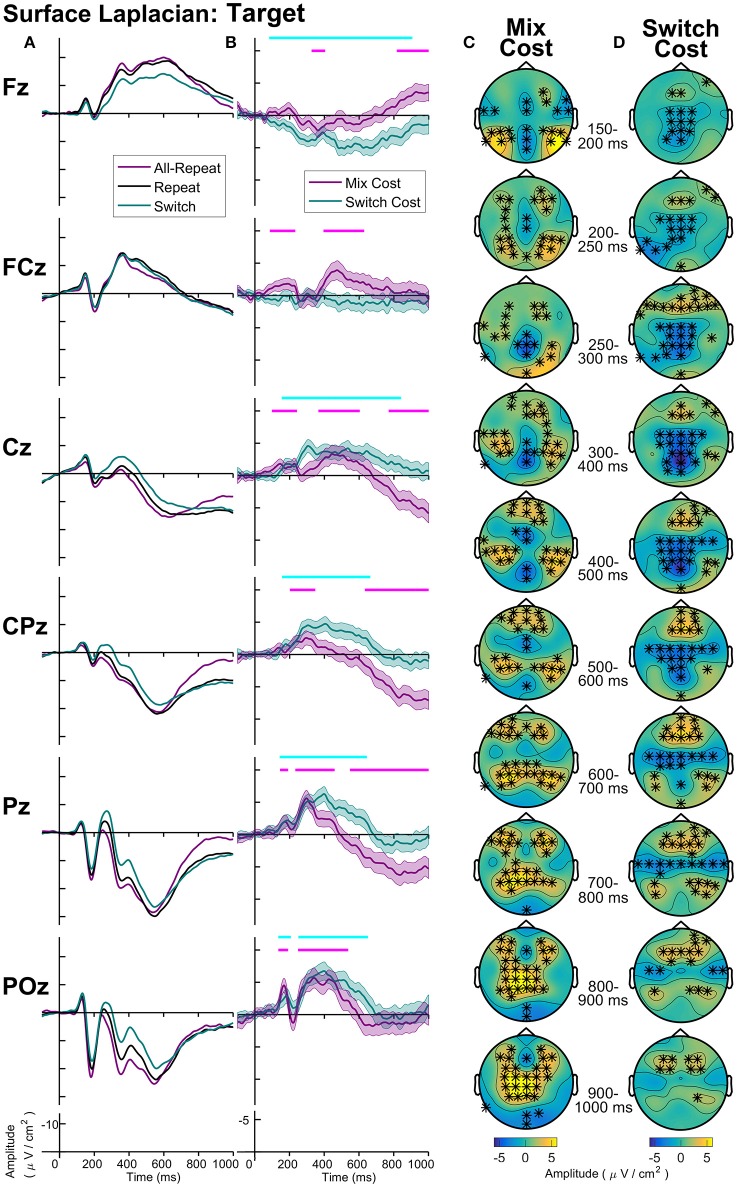
Surface laplacian target-locked: **(A)** Grand average ERPs, **(B)** Difference waveforms (Costs), Shaded 95% confidence intervals calculated for a within-subjects design (c.f., Loftus and Masson, 1994), Significant intervals of switch or mix cost (FDR α < 0.001) are shown as thin cyan and magenta bars, respectively, **(C)** Mix Cost Topographies and **(D)** Switch Cost Topographies.

Across both cue-locked and target-locked ERPs, the surface Laplacian reference scheme was sensitive to the temporal and spatial dynamics of task-switching effects that were not readily evident in other montages. Furthermore, effects that were common to all reference schemes showed a more spatially localized distribution with the surface Laplacian reference. The scalp distribution maps in Figures 4, 6 were optimized to areas of significant differences that were common across all montages. In order to focus further discussion on effects that emerge with surface Laplacian, Figures 7, 8 display these waveforms together with difference waveforms for switch cost and mixing cost at midline electrodes for cue-locked and target-locked waveforms, respectively, with shaded 95% confidence intervals calculated for a within-subjects design (c.f., Loftus and Masson, 1994), respectively. Scalp distribution maps show activity averaged across 50 ms intervals over 150–300 ms and across 100 ms intervals thereafter to examine the relative scalp distribution and time-course of switch and mixing effects.

Cue-locked mixing effects (Figure 7) represent processes required to repeat the same task within a context where task can randomly change on a trial-by-trial basis (mixed-task block) as compared to a context where the task remains the same across all trials (single-task block). Compared to all-repeat trials, repeat trials produced an increased frontocentral P2 (150–250 ms), that was accompanied by reduced activity at bilateral temporal-occipital sites (Figure 7C). A centroparietal N2-like component and a parietoccipital P3b-like component emerged for both all-repeat and repeat trials. However, the repeat cues showed a positive shift, resulting in a smaller N2 and a larger P3b, especially parietoccipitally, where it extended to the end of the CTI. From around 400 ms, repeat cues showed a slow frontal negativity and a ramp-like central negativity compared to all-repeat trials. These effects first emerged at midline sites (400–500 ms), but spread across the frontocentral scalp as CTI progressed. With one early exception, the switch cues showed a similar pattern of deviation from the all-repeat cues as the repeat cues, but the deviation was larger resulting in significant switch effects.

As seen Figure 7B, differential switch effects are characterized by a number of early rapid modulations beginning from the parietally maximal P2 and extending to circa 300 ms. Compared to repeat cues, switch cues show a larger and more prolonged parietoccipital P2 extending over time to central and bilateral frontal sources (Figure 7D). This is followed by a sharp parietal positivity with a very tight parietoccipital focus that is superimposed on a parietal N2-like component for repeat trials and that sharply dissipates by 600 ms. The sustained midline frontocentral negativity emerges around 500 ms, showing partial temporal overlap with the parietal switch positivity and extending beyond target onset. Note also the sustained bilateral parietotemporal and central positivities that are not evident at midline sites from where ERP measurements are usually derived.

Figure 8 shows the midline distribution of different trial types (Figure 8A) and difference waveforms (Figure 8B), as well as scalp distribution of mixing cost (Figure 8C) and switch cost (Figure 8D) effects after target onset. Mixing effects revealed early and transient parietal and frontal components in the first 400 ms after target, followed by a sustained, central negativity emerged from ~350–700 ms at central sites. Following this, two mixing effects emerged simultaneously: a late (~800+ ms) frontal negativity and a longer-lasting centroparietal positivity (~600+ ms). Interestingly, the scalp maps show a number of bilateral effects that are not captured at midline electrodes.

Two simultaneous signatures of the switch cost were present: A frontal switch positivity that emerged almost immediately after target onset (~80 ms) and a centroparietal switch negativity (P3b) emerging ~180 ms post-target. Interestingly, both of these switch effects were long-lasting, likely remaining until response (i.e., reliable differences were still observed as late as 900 ms after target appearance).

## Discussion

The present study compared the effects of four different spatial filters: two commonly used montages (common average and average mastoids) and two reference-free transformations (REST and surface Laplacian) on cue-locked and target-locked ERPs in a cued trials task-switching paradigm. We report three major findings. Firstly, that the commonly reported cue-locked posterior switch positivity and centroparietal mixing effects, as well as the target-locked centroparietal switch negativity were present in all four spatial transformations. However, the surface Laplacian transformation brought out a more fine-grained spatial and temporal distribution of these effects—for example, the cue-locked switch positivity was shown to have a focal, midline parietal topography compared to the broadly distributed component seen with common reference choices such as the average mastoids (Figure 4). This suggests that these commonly reported ERP effects are robust to the choice of spatial reference but that the topography (and therefore the neural generators) of these components may be misattributed when using average mastoid or common average transformations.

Secondly, the cue-locked frontal/frontocentral switch and mixing effects seen in particular reference schemes cannot be attributed to volume conduction effects. Both the frontal switch positivity and the frontocentral mixing negativity were clearly evident in the REST and surface Laplacian transformations that minimize volume conduction effects (Figure 3). This is consistent with a potential frontal generator associated with both switch and mixing costs, that has previously only been reported sporadically (e.g., Astle et al., 2008; Manzi et al., 2011; Czernochowski, 2015; Tarantino et al., 2016).

Finally, the surface Laplacian transformation revealed a rich spatiotemporal landscape associated with task-switching that was absent in all other reference choices. For example, cue-locked surface Laplacian ERPs uncovered an early emerging, transient frontal mixing positivity that resolved prior to the later canonical mixing component found in other referencing schemes. Likewise, in the first 250 ms post-cue, rapid frontal negativities and bilateral posterior positivities were associated with the switch cost—prior again to the classic parietal switch positivity. These transient effects provide new evidence of early, frontal and parietal dynamics associated with task switching. It is important to note that, given the aim of this paper, we restricted our focus on common time windows that were significant across all four transformations at three midline electrodes only. Future work focussing on a broader array of sites may reveal an even richer landscape.

Further work will be needed to identify the functional significance of these components within a task switching context. For example, both an early, cue-locked frontal N2-like and centroparietal P2-like component was seen in the surface Laplacian transformation (Figure 7). These N2-like features are typically observed in conflict paradigms relying on reactive control processes to resolve interference (see Folstein and Van Petten, 2008). An N2-like feature in a cue-locked ERP suggests that a similar action-monitoring system may be engaged early after cue onset when participants are cued to switch tasks. Perhaps such monitoring serves as a critical, stimulus-driven prerequisite step for further goal-updating processes (associated with later switch positivity).

To date, few studies have applied surface Laplacian transformations to task-switching electrophysiological data (e.g., Barceló and Cooper, 2018). When used, they are typically implemented to minimize volume conduction effects in time-frequency decompositions (e.g., Mansfield et al., 2012; Cooper et al., 2015). However, the complex pattern of spatiotemporal features seen here with the surface Laplacian transformation, suggests that these spatial filters may provide novel insights into frontoparietal network dynamics necessary for effective task switching.

For instance, it is interesting to note that the fast and slow frontal and parietal switch effects during the cue-target interval as well as the slow switch effects that emerge post-target are consistent with functional MRI evidence of frontal and parietal network involvement in task-switching. For instance, a recent ALE showed posterior parietal cortex, precuneus, inferior frontal gyrus and presupplementary motor area activations associated with switch as compared to repeat trials (Jamadar et al., 2015).

In fact, the complex temporal and spatial activation network that emerges with surface Laplacian transformations is highly consistent with evidence from time-frequency analyses showing simultaneous or partially temporally overlapping patterns of activation in different frequency bands across different scalp locations (Cooper et al., 2016) It is also consistent with evidence of distinct frontoparietal networks operating at different timepoints both in cue-target and post-target intervals (Cooper et al., 2015) and temporal shifts of theta activation across parietal and frontal regions (Cooper et al., 2017). This evidence invites future work to map out the complex frontoparietal dynamics of cognitive control processes (for recent review, see Gratton et al., 2017, 2018).

The increased spatial and temporal information provided by the surface Laplacian transformation also provides a way to examine common and distinct aspects of the neural dynamics of processes contributing to mixing and switch effects. With conventional reference montages, cue-locked mixing and switch costs appear to have common broadly distributed effects (Figure 4). Surface Laplacian shows some common components (e.g., an early parietal positivity that emerges for both mixing and switch effects, as well as distinct components (e.g., this positivity has additional later parietal components for switch costs only). Both costs again share a similar topography prior to target onset (Figure 7).

## Conclusion

The choice of spatial filter can have a strong impact on the pattern of ERPs recorded at the scalp during task-switching paradigms. All four transformations used here showed the same well-characterized ERP components that are typically seen in task-switching paradigms. However, the surface Laplacian transformation produced a much richer component landscape than conventional reference montages. The use of surface Laplacian transformation in both ERP and time-frequency analyses is recommended to increase the integration of information across these two analyses approaches of the EEG signal and to characterize EEG signatures of complex spatiotemporal networks involved in cognitive control. However, as a final cautionary note, all spatial transformations of EEG data come with their own weaknesses. As surface Laplacian is limited in its ability to model edge electrodes in the array, effects at edge electrodes need to be considered with caution.

## Author contributions

AW, PC are equal first authors; AW, methodology implementation, data analysis, idea conceptualization, over all manuscript drafting, manuscript review; PC, idea conceptualization, over all manuscript drafting, manuscript review; AC, manuscript drafting, introduction, results and discussion; MM, data analysis, manuscript drafting, manuscript review; WF, idea conceptualization, manuscript review; PM, idea conceptualization, manuscript review; FK, idea conceptualization, manuscript drafting, manuscript review.

### Conflict of interest statement

The authors declare that the research was conducted in the absence of any commercial or financial relationships that could be construed as a potential conflict of interest.
